# Video clinics versus standard face-to-face appointments for liver transplant patients in routine hospital outpatient care: study protocol for a pragmatic randomised evaluation of myVideoClinic

**DOI:** 10.1186/s13063-018-2953-4

**Published:** 2018-10-19

**Authors:** Elaine O’Connell Francischetto, Sarah Damery, James Ferguson, Gill Combes, On behalf of the myVideoClinic randomised evaluation steering group, Gill Combes, Sarah Damery, Eric Deeson, James Ferguson, Janet Jones, Foyzal Miah, Pamela Nayyar, Elaine O’Connell Francischetto, Aziz Sheikh, Katie Squire

**Affiliations:** 1 Institute of Applied Health Research, Murray Learning Centre, University of Birmingham, Edgbaston, West Midlands B15 2TT UK; 2 University Hospitals Birmingham NHS Foundation Trust, Queen Elizabeth Hospital, Mindelsohn Way, Edgbaston, West Midlands B15 2GW UK

**Keywords:** Video clinic, Liver transplant, Patient satisfaction, Randomised evaluation, Economic analysis

## Abstract

**Background:**

Video clinics, where patients can have a hospital appointment with their clinician from home, are emerging in practice, but their effectiveness is unclear. This study will evaluate whether a video clinic implemented at the University Hospitals Birmingham (UHB) NHS Foundation Trust improves patient satisfaction compared to standard face-to-face appointments for liver transplant patients.

**Methods:**

This will be a parallel, two-arm, statistician-blinded, randomised evaluation. Clinically stable liver patients at 1 to 5 years post-transplant (*n* = 180) will be randomised in equal numbers to video clinic appointments (intervention) or standard face-to-face appointments (control). The intervention group will have outpatient appointments from home via a secure video link accessed through the UHB patient portal. All patients will complete baseline questionnaires before randomisation and electronic follow-up questionnaires after each follow-up appointment during the subsequent 12 months. The primary outcome is the difference in scores between groups for three domains of patient satisfaction, namely ‘convenience of location’, ‘getting through to the office by phone’ and ‘length of time waiting’ (modified Visit-Specific Satisfaction Instrument). Secondary outcomes include quality of life (EQ-5D-5 L), costs, clinical contacts and user experience. Statistical analysis will be descriptive and performed on an intention-to-treat basis. The primary outcome will be analysed using baseline and 3-, 6-, 9- and 12-month questionnaires (according to patient follow-up appointment frequency) and comparisons made between study arms. A within-trial cost consequences analysis will be undertaken on the economic data. Patients (*n* = 8), carers/family members (*n* = 6) and health professionals (*n* = 14) will be interviewed about the experience of video clinics and the interviews will be analysed using thematic analysis.

**Discussion:**

This study will allow an in depth understanding of whether video clinics can improve patient satisfaction with their care. In addition, the intervention could save patients time and costs, removing the need to travel long distances for outpatient appointments. Video clinics may be applicable to a wide range of other clinical settings and health conditions. The study has been approved by the NHS Health Research Authority and a National Research Ethics Committee (Ref: 17/WM/0338) and research governance approval has been obtained from UHB (Ref: RRK6080).

**Trial registration:**

ISRCTN: 14093266 (25/03/2018; retrospectively registered).

**Electronic supplementary material:**

The online version of this article (10.1186/s13063-018-2953-4) contains supplementary material, which is available to authorized users.

## Background

Increasing the use of technological interventions to improve patient care is becoming more prominent in national policy, especially for people with chronic conditions [1–4], with the UK Government having set a target to offer digital services, including electronic consultations, to 95% of general practice patients by 2020 [2]. Emerging interventions have included online patient access to health records, computerised therapy, video conferencing and home sensors [5–8]. The 2016 Wachter Review on Health Information Technology to Improve Care in England concluded that the English secondary care sector is ready for a national digital strategy to help create a user-centred infrastructure that is high quality and manageable [4].

Video clinics (also known as tele-consultations, virtual clinics, virtual consultations, e-consultations, remote video visits, skype consultations or videoconferencing) have started to emerge in the evidence base for different patient groups, including patients in primary care [5, 9, 10], secondary care [5, 10, 11] and integrated/joint care [10, 12, 13]. In secondary care, some studies have used videoconference technology for remote consultation [5, 11, 14, 15]. However, video clinic studies in secondary care have typically had small sample sizes and differ in their findings. For example, one study of video clinics in orthopaedics found higher rates of patient satisfaction in the intervention group [14], whilst another found similar levels of patient satisfaction in both intervention and control groups [15]. The evidence is also unclear about the cost-effectiveness of telehealth services [16]. For example, studies focusing on teledermatology have found that remote consultation costs were less than those of conventional medicine when large geographical distances exist between specialist care providers and patients [17], and that teledermatology is not cost-effective for patients travelling smaller distances [18]. In contrast, a large, well-designed UK study focusing specifically on the effectiveness of joint consultation between patients, General Practitioners (GPs) and hospital specialists found that joint consultations can significantly increase patient satisfaction with treatment, but were associated with higher costs over 6 months [13, 19].

Numerous examples of video clinic interventions have emerged in clinical practice in recent years in the UK [20, 21] and America [22]. However, current evidence does not clearly show the benefits of video clinics in secondary care and there is a lack of large well-designed studies assessing the effectiveness of video clinics to facilitate outpatient appointments in patients’ homes. There is an ongoing need for randomised controlled e-health studies and high-quality evaluations that include research on patient perspectives, satisfaction and costs [11, 23–25].

### Video clinics for liver transplant patients at University Hospitals Birmingham (UHB) NHS Foundation Trust

The UHB NHS Foundation Trust Liver and Hepato-Pancreato-Biliary (HPB) Unit is one of the largest in the UK, performing over 250 liver transplants annually. Once patients are considered clinically stable (usually 1 year post-transplant), they must attend the hospital regularly for ongoing monitoring (follow-up appointments every 3 or 6 months). As one of only six specialist adult liver transplant centres in the UK, the Liver and HPB Unit covers an extensive catchment area, and many transplant patients travel large distances to attend follow-up appointments. In collaboration with patients and clinicians, UHB has developed a video clinic tool that is accessible via the Trust patient records portal (myhealth@QEHB). The portal was developed by the Technical Development and Informatics team at the Trust, and is currently used by over 7000 patients across 40 clinical specialties. It allows patients to remotely access some of their clinical information, including letters, laboratory results, email correspondence with their consultant and GP referrals, and patients can co-manage their treatment plans with the relevant clinicians. Patients can also view appointments, receive reminders, upload/share files on the system and interact with other patients to create their own peer-support networks.

Of the 3000 liver transplant patients considered stable at any one time at UHB, approximately 1000 currently use myhealth@QEHB and the Liver and HPB Unit is introducing video clinic appointments (termed myVideoClinic) via the portal for these patients. Video clinics will allow routine 3 or 6 monthly hospital appointments to be carried out remotely via a secure video and/or voice link between the patient and their hospital consultant. MyVideoClinic will also allow patients to pre-specify three questions/topics that they would like to discuss during their video clinic, and an audio recording of the video clinic will be made available in their patient record after their appointment. Patients using the video clinic service will need to have the relevant clinical testing for each appointment (blood tests, weight and blood pressure measurements) carried out at a GP practice or dialysis centre local to their home and the test results must be uploaded to the portal prior to each myVideoClinic appointment.

Audit data collected from one of the UHB liver clinics in early 2017 showed that, although satisfaction with outpatient care in liver transplant patients is generally high (as measured by the modified Visit-Specific Satisfaction Instrument, VSQ-9) [26], satisfaction was significantly lower in the specific VSQ-9 domains of ‘convenience of location’, ‘getting through to the office by phone’ and ‘length of time waiting’ when these were compared to other domains (*n* = 83; *p* < 0.0001). Perceived convenience, ease of contacting the hospital and waiting times are therefore all aspects of patient satisfaction that could potentially be improved by offering video clinics and introducing flexibility in how patients receive their post-transplant outpatient care. MyVideoClinic may also lower patient and secondary care costs.

This protocol outlines a pragmatic, two-armed, parallel group, statistician-blinded randomised evaluation designed to assess the effectiveness of providing video clinics as an alternative to standard face-to-face consultations in delivering routine follow-up care for clinically stable liver transplant patients.

## Methods/Design

**Fig. 1:**
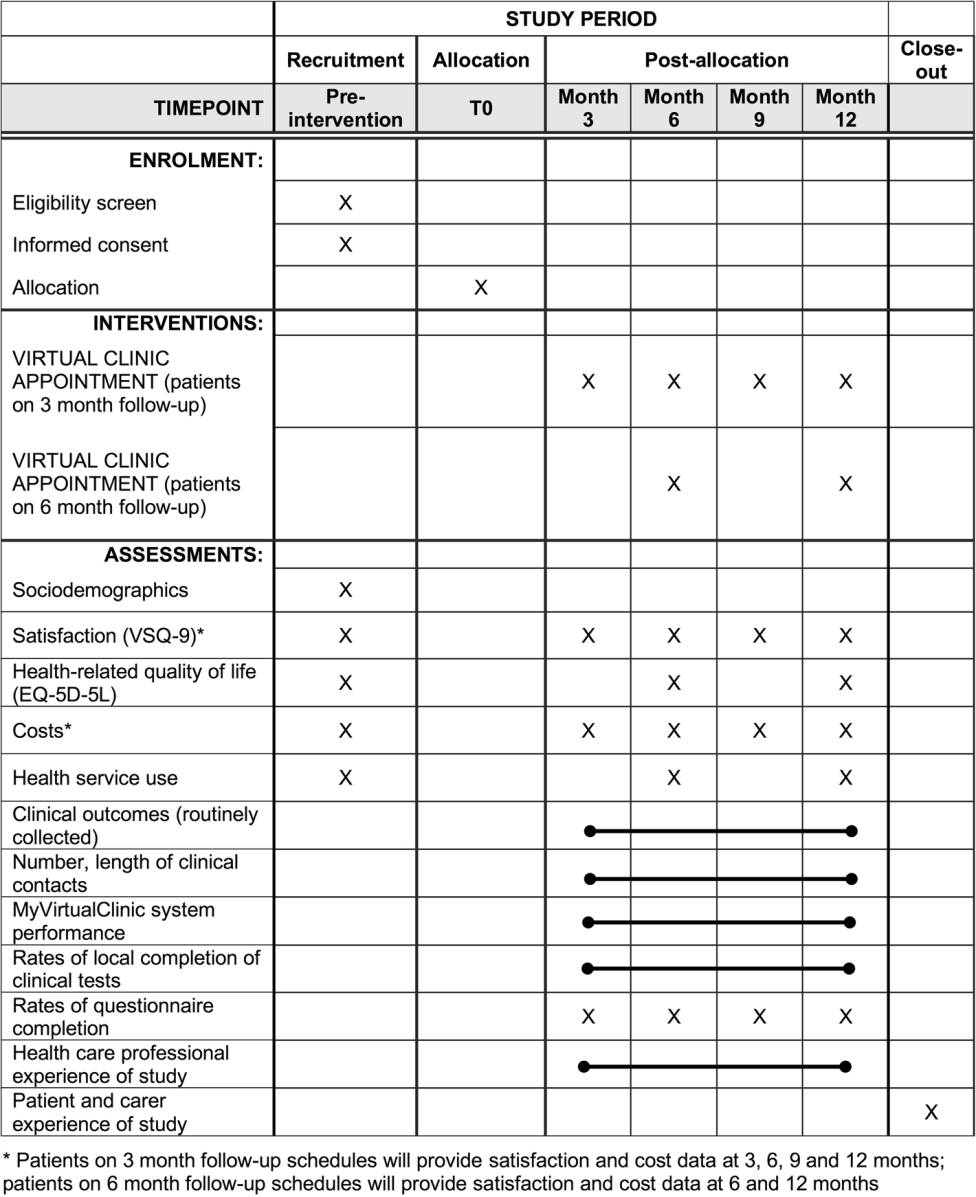
SPIRIT Schedule of enrolment, interventions, and assessments

The protocol structure and content follows the SPIRIT guidelines (see Additional file 1 for checklist) [27]. A completed SPIRIT figure shows the schedule of recruitment, interventions and assessments (Fig. 1).

### Aims

The primary aim is to assess whether the option of myVideoClinic can increase patient satisfaction in the VSQ-9 domains of ‘convenience of location’, ‘getting through to the office by phone’ and ‘length of time waiting’ compared to standard care (face-to-face consultations) for clinically stable liver transplant patients.

The secondary aims are to:

1. Assess the impact of myVideoClinic on patient satisfaction in the other six VSQ-9 domains
2. Assess whether myVideoClinic can improve patient health-related quality of life (HRQoL)
3. Evaluate the patient and secondary care costs associated with myVideoClinic
4. Evaluate health service use and the number (and length) of clinical contacts
5. Review the technical performance of the video clinic
6. Review the feasibility of clinical testing being performed locally at the patient’s home
7. Review questionnaire completion rates
8. Evaluate the effect of the video clinic on travel time
9. Explore patients’ and carers’ opinions and experiences of myVideoClinic
10. Explore health professionals’ opinions and experiences of myVideoClinic

### Participants

Patients will be recruited from four outpatient liver clinics at the Queen Elizabeth Hospital Birmingham (QEHB) (primary sclerosing cholangitis, primary biliary cholangitis, alcoholic liver disease and autoimmune hepatitis). Each year, approximately 267 patients at 1 to 5 years post-transplant attend routine hospital appointments at QEHB.

#### Participant inclusion criteria

1. Have had a liver transplant at least 1 year and no more than 5 years prior to study baseline
2. Aged 18 or over
3. Considered clinically stable by their consultant
4. Have access to myhealth@QEHB (or agree to sign up)
5. Able to arrange for clinical testing (blood tests, weight and blood pressure) to be undertaken locally at a GP practice or dialysis centre and the results uploaded onto myhealth@QEHB prior to the myVideoClinic appointment
6. Have access to a computing device (e.g. desktop computer or laptop) running an operating system compatible with the video clinic software, as well as a camera and internet connection to allow access to myVideoClinic from home
7. Able to consent to participate in the study

#### Participant exclusion criteria

1. Unable to speak and/or read English
2. Unable to comply with study follow-up procedure (completion of electronic questionnaires)
3. Involvement in another research study or clinical trial involving ongoing questionnaire completion

### Recruitment and consent

Prior to each clinic where recruitment is taking place, a member of the clinical team will screen the day’s clinic list to identify eligible patients on the basis of time since transplant and age (inclusion criteria 1 and 2). Patients who meet these criteria will have the study introduced to them at the end of their routine outpatient appointment and their further eligibility assessed against inclusion criteria 3 to 7. The consultant will give eligible patients a Participant Information Sheet and those interested in study participation will have the opportunity to discuss the study with a member of the University of Birmingham research team. The research team will take written informed consent during the clinic from patients who wish to participate and the latter will complete a baseline questionnaire prior to randomisation. Patients who require more time to consider participation will be able to take the study information away and will be contacted by telephone within 7 days by a member of the research team. If a patient agrees to take part over the telephone, a consent form and baseline questionnaire will be posted to them along with a reply-paid envelope for return of the completed documents to the University of Birmingham. Once recruited, participants will stay in the study for 12 months. This will equate to between two and four follow-up appointments since the participants’ usual appointment schedule and frequency will continue to be defined by clinical need regardless of group allocation.

If a patient does not wish to participate in the study, their reason for refusal will be recorded, along with routinely collected information (age, sex and clinic attended). To further understand reasons for participants not taking part, a selection of non-participating patients (*n* = 4) will be invited to take part in a short semi-structured interview (see qualitative study section).

### Randomisation and blinding

After giving consent and completing the baseline questionnaire, participating patients will be randomised in a 1:1 ratio to either the intervention (myVideoClinic) or control (standard care) arm of the study using the GraphPad online randomisation tool [28]. Participants will be randomised by the member of the research team who has taken participant consent. Patients randomised to the myVideoClinic group will be registered on the myVideoClinic system and given printed information on how to access an online training tool that includes instructions on how to use the video clinic. Patients from both study arms will be registered on the myhealth@QEHB data collection system. Due to the nature of the intervention, blinding of study participants or clinical staff administering myVideoClinic will not be possible. The statistician analysing the primary outcome data will be blinded to each participant’s study group for the intention-to-treat analysis.

### Intervention group

Patients randomised to the myVideoClinic group will be sent appointments for their video clinic through the post and will receive standard text reminders about their appointment. Patients will be required to have their clinical tests (blood test, weight and blood pressure) carried out locally at their GP practice or at a nearby dialysis centre and make the test results available through myhealth@QEHB before each video clinic appointment. At each appointment, patients will log in to myhealth@QEHB and speak to their consultant via an embedded video link or voice call (depending on their internet bandwidth). Video clinics will use the secure system Vidyo as the videoconferencing platform. In addition to having their follow-up appointment via myVideoClinic, the system will allow patients to submit up to three questions or topics prior to their appointment that they would like to discuss with their clinician, and patients will be able to access an audio recording of the video clinic from their patient record after each appointment.

If there are any technical issues with myVideoClinic during a video appointment, the consultant will telephone the patient to finish the appointment and schedule a face-to-face appointment if necessary. All instances where appointment rescheduling has occurred will be recorded by the clinician. If patients in the intervention group experience any technical problems with myVideoClinic during the study, they are advised to contact the myhealth@QEHB support team.

### Control group

Patients randomised to the control group will receive standard face-to-face care at the hospital and will not be offered video clinics. They will receive standard written notifications of the date and time of their appointment and routine text reminders, and will also complete their clinical tests in hospital on the day of their appointment, the results of which will be reviewed by the clinician after each patient’s appointment.

#### Crossover between study arms

Patients allocated to the intervention group will be made aware that the intervention is the option of having a video clinic as an alternative to face-to-face appointments and that they can ask to come to the hospital for a standard face-to-face appointment if they wish. They will still be able to have a video clinic for their next appointment as long as they complete their clinical tests locally. Clinical staff will also be able to change patients from using myVideoClinic to a standard face-to-face appointment if (1) the patient has requested this, (2) there is a clinical need to see the patient face-to-face, or (3) if a consultant has not seen the results of pre-appointment clinical testing for a myVideoClinic patient for two successive appointments. Staff will be asked to record any reasons for changing patients from myVideoClinic to standard care. The attendance to a face-to-face appointment by a patient assigned to the video clinic group will not be treated as study withdrawal as we have allowed for patients in the video clinic group to crossover between face-to-face appointments and video clinics for clinical reasons and because pilot work has shown that patients have a preference for flexibility. Crossover between study arms will be monitored, recorded and reported in both interim and main study analyses and will be discussed with the study’s independent monitoring group if crossover exceeds 60%.

### Outcome measures and data collection

Study outcomes and methods of data collection are summarised in Table 1.

**Table 1:** Outcome measures, data collection instruments and format

Outcome measure	Data collection instrument	Format
Primary outcome		
(Change in) satisfaction in the VSQ-9 domains of ‘convenience of location’, ‘getting through to the office by phone’ and ‘length of time waiting’	Modified Visit-Specific Satisfaction instrument (VSQ-9) [26] within patient questionnaire	Baseline: paper At the following timepoints, after each appointment, in electronic or paper^a^ format: 3 months (if applicable) 6 months 9 months (if applicable) 12 months
Secondary outcomes		
Patient-reported health-related quality of life	EQ-5D-5 L [31] within patient questionnaire	Baseline: paper 6 months: electronic or paper 12 months: electronic or paper
Patient satisfaction scores in the other six VSQ-9 domains	VSQ-9 [26] within patient questionnaire	Baseline: paper At the following timepoints, after each appointment, in electronic or paper^a^ format: 3 months (if applicable) 6 months 9 months (if applicable) 12 months
Routinely collected clinical outcomes	Patient records	Routinely collected metrics
Number and length of clinical contacts, and instances of appointment non-attendance	Length of appointment, phone calls, myhealth@QEHB correspondence, crossover between study arms	Routinely collected metrics Clinician reporting Case report forms
Health service use	Patient questionnaire	Baseline: paper At the following timepoints, after each appointment, in electronic or paper^a^ format: 6 months 12 months
Costs (NHS perspective and societal perspective)	Patient questionnaire and routinely collected data	Baseline: paper At the following timepoints, after each appointment, in electronic or paper^a^ format: 3 months (if applicable), 6 months 9 months (if applicable) 12 months Routinely collected metrics
MyVideoClinic system performance	Failed appointments, service outages, telephone consultations	Routinely collected metrics
Clinical tests performed locally prior to video clinic appointment	Blood tests, blood pressure and weight	Case report form completed by clinician
Patient, carer and clinician experience	Semi-structured interviews	Semi-structured interviews
Questionnaire completion rates	Number of questionnaires completed	Baseline: paper At the following timepoints, after each appointment, in electronic or paper^a^ format: 3 months (if applicable) 6 months 9 months (if applicable) 12 months

^a^If any patients experience technical issues providing data electronically, paper versions of the relevant questionnaires and reply-paid envelopes will be posted directly to their home address for completion

#### Primary outcome measure

The combined satisfaction score for three domains of the modified VSQ-9 (convenience of location, getting through to the office by phone and length of time waiting) is the primary outcome for this study [26]. The VSQ-9 has been validated and used in previous studies conducted in different health settings [29], and an earlier version of this questionnaire (SVQ13) was used in a randomised controlled trial of virtual outreach in a UK context [30]. The VSQ-9 asks participants to rate their satisfaction with various aspects of their clinic appointment on a 5-point scale (poor, fair, good, very good, excellent). Scores are then transformed into a 0–100 linear scale, where higher scores denote higher levels of satisfaction. VSQ-9 data will be collected at baseline and after every video clinic or face-to-face appointment via patient questionnaires. A difference of 10 points between the intervention and control groups in the three selected domains of the VSQ-9 at study end (12 months) will be taken as clinically significant [30].

#### Secondary outcome measures

Secondary outcomes include satisfaction scores for the other six domains of the VSQ-9 (collected at baseline and after each appointment); routinely collected clinical data from patient records; number and length of clinical contacts; health service use; patient and secondary care costs associated with video clinics and face-to-face appointments; patient travel requirements; whether patients in the intervention group have been able to have clinical tests done locally before their appointment; post-appointment questionnaire completion rates; patient, carer and clinician experience; technical data on myVideoClinic system performance; and HRQoL.

HRQoL data will be collected at baseline, 6 months and study end using the EQ-5D-5 L [31], which assesses patient functioning and wellbeing with respect to mobility, self-care, usual activities, pain/discomfort and anxiety or depression. Levels of difficulty in each of these areas are reported using a five-point ordinal scale, the combination of which gives each patient a health profile. Although it is recommended that quality of life for liver transplant patients should be measured using both a generic quality of life questionnaire and a disease-specific instrument, a precise and reliable measure of quality of life for liver transplant patients could not be identified [32, 33]. The EQ-5D-5 L is straightforward to complete, can be administered in different ways, can be used as a utility measure and has been used in previous studies with liver transplant patients [33, 34].

### Data collection time points

**Fig. 2:**
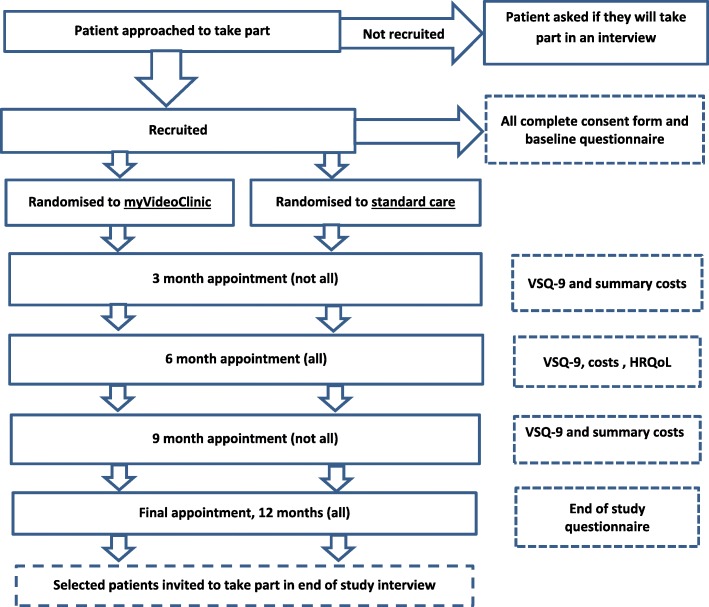
Time points for data collection

Figure 2 summarises the time points for data collection for patients in the intervention and control groups.

#### Baseline

All patients who have consented to participate in the study will complete baseline questionnaires before being told the outcome of randomisation. Baseline questionnaires will record the following information:

- Sociodemographics (e.g. home postcode, sex, age, ethnicity, employment status)
- Clinical information (e.g. diagnosis, comorbidities, time since transplant)
- Current computer usage
- Patient satisfaction (modified VSQ-9)
- HRQoL (EQ-5D-5 L)
- Costs (e.g. healthcare use, travel and personal expenses associated with appointment attendance)

#### Follow-up

Patients will complete patient satisfaction and summary cost questionnaires after each myVideoClinic or standard hospital appointment. For participants on a 3-monthly follow-up schedule, data will be collected at baseline and at 3, 6, 9 and 12 months. For participants on a 6-monthly follow-up schedule, data will be collected at baseline and at 6 and 12 months. For all participants, the 6- and 12-month questionnaires will collect full data on satisfaction, costs (e.g. health service use, travel and personal expenses), IT issues and HRQoL. Patients who also have appointments at 3 and 9 months after baseline will complete shorter questionnaires at these time points, covering satisfaction and costs only. Questionnaires will be sent to patients in both study arms electronically through the myhealth@QEHB system no more than 7 days after each appointment.

### Qualitative study

In accordance with Medical Research Council guidelines for evaluating complex interventions [35], an embedded qualitative study will explore patient, carer/family member and healthcare professionals’ experiences and perceptions of myVideoClinic. Semi-structured interviews will be undertaken with the following groups:

1. Patients randomised to the standard care group (*n* = 4)
2. Patients randomised to myVideoClinic (intervention) group (*n* = 8)
3. Carers/family members of patients in the intervention and control groups (*n* = 6)
4. Patients who did not wish to take part in the randomised evaluation (*n* = 4)
5. Healthcare professionals involved in the care of the intervention patient group (*n* = 14)

#### Patients

Patients randomised to the intervention group will be stratified according to their degree of engagement with the intervention (patients completing 50% or fewer of their scheduled video clinic appointments vs. those completing more than 50%). Patients will be purposively sampled to ensure diversity on key variables such as age and sex. Selected patients will be invited to take part in a semi-structured interview to explore their opinions or experiences of using the myVideoClinic and any suggestions on how it could be improved at the end of their involvement in the study (*n* = 8). Purposively sampled patients in the control group (*n* = 4) will also be interviewed about their experiences of participating in the study.

#### Carers/family members

Carers/family members of patients being interviewed at the end of the study (*n* = 6) will be invited to take part in an interview to explore their experiences of caring for/living with a patient in the video clinic study and any impact of study participation on them.

#### Patients who do not wish to take part in the randomised evaluation

A small number of eligible patients (*n* = 4) who decline participation in the study will be interviewed to discuss their reasons for not taking part. This will provide important information that may inform the subsequent rollout of video clinics to other clinical specialties or sites if the intervention is found to be effective.

#### Health professionals

A purposive sample of 14 health professionals (clinicians who administer consultations, *n* = 8; staff who administer clinical tests at the hospital, *n* = 2; primary care staff administering clinical tests locally, *n* = 4) will be interviewed at the end of the study. Interviews will explore staff experiences of the myVideoClinic system, including any perceived advantages (e.g. cost, time and quality of care) and disadvantages (e.g. lack of physical examination). Interviews will provide information on how best to engage and support health professionals in adopting video clinics within routine care if the intervention is considered effective.

### Sample size and study power

**Fig. 3:**
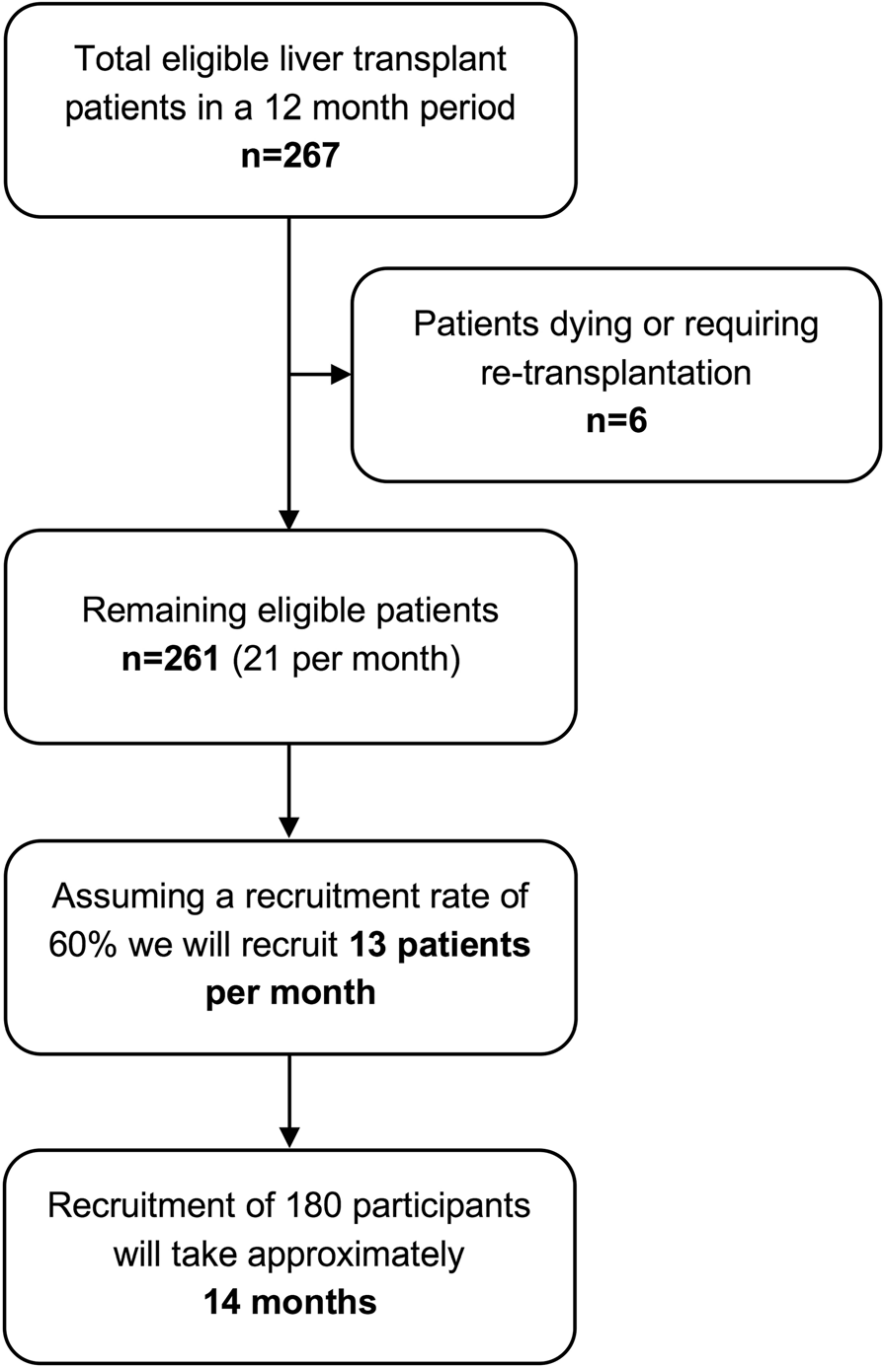
Recruitment rate

The sample size for the study has been determined by the effect size needed to detect a clinically significant (10 point) difference between intervention and control groups in the primary outcome measure (patient satisfaction as measured on three domains of the VSQ-9). Assuming a normal distribution of satisfaction scores, a sample size of 62 patients per group would be sufficient to detect a 10-point difference in VSQ-9 scores between groups at 80% power and an alpha of 0.05. Over a 12 month period, an estimated 267 eligible patients will be available for recruitment to the study from the four liver clinics of interest. Annually, approximately 1% of liver transplant patients at UHB undergo a re-transplant and 2% die. Thus, around 21 patients per month will be eligible for recruitment. Audit data collected from one liver clinic in early 2017 suggest that approximately 60% of eligible patients will be willing to participate in the study (13 per month). A previous randomised controlled study of video clinics in diabetes care reported an attrition rate of 30% [12]. Applying this attrition rate to our patient sample, and allowing for participant withdrawals, missing data and losses to follow-up, the required sample size for the study is 90 per group (*n* = 180 total). Recruiting at a rate of 13 patients per month, we anticipate recruitment to take approximately 14 months in total (Fig. 3).

### Statistical analysis

An interim analysis will be undertaken once 25% of the target recruitment sample have completed two appointments. This interim analysis will focus on crossover from the intervention to control groups due to patients being unable to complete their clinical testing locally. The findings from this interim analysis will be discussed with the study’s independent monitoring group as high rates of crossover have implications for the feasibility of the intervention and will reduce study power to detect significant differences between intervention and control arms in the outcomes of interest. The main data analysis will be undertaken on an intention-to-treat basis, although an unblinded per protocol analysis will also be performed for the primary outcome measure to assess whether similar results are obtained with regard to any statistically significant findings. It is acknowledged that younger patients may potentially find the intervention more beneficial due to greater familiarity with technology, so age will be controlled for and a sensitivity analysis will assess whether age has an independent effect on outcomes.

The characteristics of study participants at baseline will be summarised descriptively, with comparison of proportions tests used to assess any differences between intervention and control groups on the basis of sociodemographic and clinical characteristics. Analysis of the primary outcome measure (difference between groups in scores in three domains of the VSQ-9) will use independent *t* tests if data are normally distributed. If data are not normally distributed, the Mann–Whitney U test will be used. For patients with missing end-of-study VSQ-9 data, their most recently collected data will be used in the analysis. If participant numbers allow, subgroup analysis will assess patient satisfaction scores adjusting for the level of engagement that intervention patients had with myVideoClinic (i.e. those with 6 months or less of video clinic appointments vs. those with more than 6 months of video clinic appointments).

Secondary outcome measures will be analysed descriptively where the same data have been collected from patients in both study arms (e.g. health service use). Where secondary outcome data are relevant to the intervention group only (e.g. the incidence of technical issues during myVideoClinic appointments), these will be tabulated and basic statistical analyses performed (numbers, percentages, means/medians etc.). Attrition/withdrawal rates and rates of questionnaire completion will be analysed for both study arms and overall. Analysis of HRQoL data will follow the recommendations in the EQ-5D-5 L User Guide [34]. Health state valuations will be calculated for each profile based on pre-calculated scoring coefficients. Changes in HRQoL between baseline and study end will be assessed for each patient and both descriptive and comparative analyses will be performed to compare HRQoL between participants in the intervention and control groups.

#### Economic analysis

A within-trial cost consequences analysis (CCA) will be undertaken. CCA allows consideration of a number of outcome measures alongside costs, rather than a pre-defined single outcome measure, as would be the case in a cost utility analysis using quality-adjusted life years. Base-case analysis will be conducted from the NHS perspective, with additional analyses from the societal perspective. The NHS perspective will include health service resource utilisation in terms of provider costs, commissioner costs (prices) and costs that are sometimes paid for by patients (e.g. travel). Analysis will also include data on NHS resource use for patient appointments (e.g. number of face-to-face appointments, myVideoClinic calls, telephone calls, emails) and costs of clinical tests. The societal perspective will include non-NHS costs, such as patients’ out-of-pocket expenses (e.g. travel costs, car parking, childcare costs), and wider costs such as productivity loss due to absence from work. The CCA will include a sensitivity analysis to model the cost consequences of a number of different scenarios, whereby patients take up different numbers of appointments and different proportions of patients take up myVideoClinic. Including both the NHS and societal perspectives will provide a full picture of how video clinics affect the financial burden on both the NHS and patients and may help to inform the development of new payment currencies for patient management. This is important in terms of the acceptability of myVideoClinic to the provider and commissioners because any move away from traditional face-to-face appointments risks a loss of provider income, unless locally agreed funding arrangements are implemented, which reflect the intervention’s aims to promote more efficient use of resources.

#### Qualitative data

All interviews will be audio-recorded, transcribed verbatim and analysed using thematic analysis. The thematic analysis will be undertaken in line with the methodology outlined by Braun and Clarke [36]; 10% of the transcripts will be independently coded by a second researcher and compared, discussed and amended prior to defining the themes. Qualitative data will be analysed as data is collected to allow emerging themes to be explored in later interviews.

### Safety

The overall clinical responsibility and welfare of patients involved in the study will remain with the consultant who is providing their care and the clinical care the patient receives will not change. Any serious adverse events or adverse events reported through myhealth@QEHB or during interviews will be reported to the clinical lead immediately by the informatics team or researcher. The clinical lead will then notify the consultant responsible for the care of the patient to ensure this is dealt with immediately.

### Data monitoring

The research team and study steering group will be accountable throughout the study to an independent monitoring group, which will meet every 6 to 9 months. The independent monitoring group members will participate in discussions regarding study progress, review an unblinded report on interim study data (e.g. recruitment rates and interim analysis results) and offer independent advice to the study team.

### Data management and participant confidentiality

All patient information will remain confidential throughout the study as required by ethical and research governance approvals. At the initial eligibility screening stage, an eligibility case report form will be completed by the patient’s consultant, which records patient initials, age and sex. Once a patient has given written consent to be involved in the study, a randomisation case report form will be completed by a member of the research team and the participant assigned a unique, anonymised identifier. Members of the research team will have access to patient-identifiable data during the study, but this will only be after participants have given consent. All electronic files will be held at the University of Birmingham on password-protected secure university servers to which only members of the research team will have access. Paper copies of research data or study documentation will be held securely in a locked archive in a locked office in a swipe-card restricted area of the University of Birmingham Institute of Applied Health Research.

## Discussion

The myVideoClinic study is patient centred and has been designed with input from patients, clinicians, researchers and IT staff. It is anticipated that myVideoClinic will utilise technology to improve patient satisfaction with their care. In addition, myVideoClinic could provide cost and time savings for patients, removing the need to travel long distances for outpatient appointments. Findings from this research could be applicable to a wide range of settings that could use video clinics. The embedded qualitative study will allow the views of patients, carers/family members and healthcare professionals on the video clinic to be explored. These findings will allow a more in-depth understanding of how the video clinic was beneficial or how it could be improved for future practice.

## Study status

Study recruitment commenced on March 12, 2018, and is anticipated to continue until May 2019.

## Additional file

Additional file 1: SPIRIT 2013 33-item checklist. (DOC 121 kb)

## Abbreviations

CCA: cost consequences analysis
GP: General Practitioner
HPB: hepato-pancreato-biliary
HRQoL: health-related quality of life
QEHB: Queen Elizabeth Hospital Birmingham
UHB: University Hospitals Birmingham
VSQ-9: Visit-Specific Satisfaction Instrument
